# Imaging markers of cerebral amyloid angiopathy and hypertensive arteriopathy differentiate Alzheimer disease subtypes synergistically

**DOI:** 10.1186/s13195-022-01083-8

**Published:** 2022-09-30

**Authors:** Ting-Bin Chen, Wei-Ju Lee, Jun-Peng Chen, Shiang-Yu Chang, Chun-Fu Lin, Hung-Chieh Chen

**Affiliations:** 1 Department of Neurology, Neurological Institute, Taichung Veterans General Hospital, No.1650, Sect. 4, Taiwan Boulevard, Taichung, 40705 Taiwan; 2 Dementia Center, Taichung Veterans General Hospital, Taichung, Taiwan; 3 Center for Geriatrics and Gerontology, Taichung Veterans General Hospital, Taichung, Taiwan; 4 School of Medicine, National Yang-Ming Chiao Tung University, Taipei, Taiwan; 5 Biostatistics Task Force of Taichung Veterans General Hospital, Taichung, Taiwan; 6 Division of Neuroradiology, Department of Radiology, Taichung Veterans General Hospital, No.1650, Sect. 4, Taiwan Boulevard, Taichung, 40705 Taiwan

**Keywords:** Biomarkers, Lacunes, Microbleeds, Small vessel disease, White matter hyperintensities

## Abstract

**Background:**

Both cerebral amyloid angiopathy (CAA) and hypertensive arteriopathy (HA) are related to cognitive impairment and dementia. This study aimed to clarify CAA- and HA-related small vessel disease (SVD) imaging marker associations with cognitive dysfunction and Alzheimer disease (AD) subtypes.

**Methods:**

A sample of 137 subjects with clinically diagnosed late-onset AD identified from the dementia registry of a single center from January 2017 to October 2021 were enrolled. Semi-quantitative imaging changes (visual rating scale grading) suggestive of SVD were analyzed singularly and compositely, and their correlations with cognitive domains and AD subtypes were examined.

**Results:**

Patients with typical and limbic-predominant AD subtypes had worse cognitive performance and higher dementia severity than minimal-atrophy subtype patients. Deep white matter hyperintensity (WMH) presence correlated inversely with short-term memory (STM) performance. The three composite SVD scores correlated with different cognitive domains and had distinct associations with AD subtypes. After adjusting for relevant demographic factors, multivariate logistic regression (using minimal-atrophy subtype as the reference condition) revealed the following: associations of the typical subtype with periventricular WMH [odds ratio (OR) 2.62; 95% confidence interval (CI), 1.23–5.57, *p* = 0.012], global SVD score (OR 1.67; 95%CI, 1.11–2.52, *p* = 0.009), and HA-SVD score (OR 1.93; 95%CI, 1.10–3.52, *p* = 0.034); associations of limbic-predominant subtype with HA-SVD score (OR 2.57; 95%CI, 1.23–5.37, *p* = 0.012) and most global and domain-specific cognitive scores; and an association of hippocampal-sparing subtype with HA-SVD score (OR 3.30; 95%CI, 1.58–6.85, *p* = 0.001).

**Conclusion:**

Composite SVD imaging markers reflect overall CAA and/or HA severity and may have differential associations with cognitive domains and AD subtypes. Our finding supports the possibility that the clinical AD subtypes may reflect differing burdens of underlying CAA and HA microangiopathologies.

**Supplementary Information:**

The online version contains supplementary material available at 10.1186/s13195-022-01083-8.

## Background

Alzheimer’s disease (AD) has a heterogeneous presentation and several subtypes of AD have been distinguished based on clinical and neuropathological features [1–4]. Advances in postmortem biomarker research, neuroimaging, and in vivo biomarker methods have yielded data supporting these biologically defined subtypes of AD [1, 5, 6]. The neurobiological profiles of AD subtypes cannot be distinguished based solely on β-amyloid pathology or distribution of β-amyloid retention on positron emission tomography [7]. Because the subtypes have differing demographic, neuropathological, neuropsychological, and neuroimaging correlates as well as differential biomarker profiles and clinical trajectories over time [1, 5, 6, 8, 9], it is possible that their particular etiologies may underlie patient variance in responsiveness to trial anti-amyloid medications. Hence, clarifying the changes to brain structure, neuropathology, and cognition that are characteristic of each subtype may explain, to some extent, known AD heterogeneity and lead to improved predictability of how individual patients are likely to respond to treatments.

AD pathology rarely occurs in isolation and the number of co-pathologies tends to increase with age [10, 11]. The added burden of other vascular and degenerative pathologies—such as cerebrovascular lesions, Lewy body-related pathology, and limbic TAR-DNA binding protein-43 (TDP-43) deposition—can affect the clinical picture of AD and may accelerate disease progression by lowering the threshold of AD pathology that produces clinical symptoms [5]. In particular, small vessel disease (SVD) may play a central role in AD heterogeneity [12, 13]. SVD shares risk factors with AD, namely hypertension, diabetes mellitus, smoking, hyperlipidemia, and apolipoprotein E ε4 allele presence, and the presence of SVD is associated with increased risk for stroke, cognitive decline, and dementia in the older adults [14, 15]. Various features of SVD pathologies reflective of cerebral amyloid angiopathy (CAA) and hypertensive arteriopathy (HA) are prevalent in AD brains [13, 16]. CAA and HA are distinct microangiopathies with differentiated pathophysiologies, clinical significances, and prognoses [17]. There are well established associations of CAA and HA with distributions of magnetic resonance imaging (MRI) markers, including cerebral microbleeds (CMBs), white matter hyperintensities (WMH), lacunar infarcts, and perivascular space enlargements (PVSEs) that are helpful for differentiating between CAA and HA neuroradiologically [18]. CAA is characteristic of progressive β-amyloid deposition in small cortical vessels overlying the leptomeninges and gray-WM junction, while HA primarily affects the small perforating arteries of deep gray nuclei and deep WM [19]. Thus, the topographic distribution of CAA imaging markers tends to be in lobar regions or centrum semiovale (CSO), while that of HA markers tends to be in deep/infratentorial regions or basal ganglia (BG) [20, 21]. In combination, these markers constitute clinically salient MRI signatures of SVD burden, and composite SVD scores have been shown to associate inversely with performance on cognitive, functional ability, gait, and balance tests [22–25]. Based on the common coexistence of CAA and HA and possible interactions between them, it has been suggested that both may involve impairments of the blood–brain barrier (BBB) and glymphatic clearance system [19]. However, their pathophysiological and clinical impacts on AD subtypes are not clear.

The aim of this study was to investigate the differences of SVD imaging markers across AD subtypes based on patterns of brain atrophy on MRI. For this purpose, we adopted the following four previously established AD subtypes based on brain atrophy revealed by MRI: typical, limbic-predominant (LP), hippocampal-sparing (HS), and minimal-atrophy (MA) subtypes [1, 8, 26–28]. SVD load evident on MRI was semi-quantified based on three validated scales used to assess global SVD, CAA-specific burden, and HA-specific burden [22, 23, 29, 30]. The data obtained were used firstly to characterize cognitive and imaging variables across the four AD subtypes and secondly to determine whether SVD imaging markers correlate with cognitive performance and AD subtype. The findings may have both research and clinical applications. The present study may help to elucidate complex microangiopathic mechanisms underlying the development of AD subtype-distinct atrophy patterns in outpatients with a clinical diagnosis of late-onset AD based on the criteria proposed by the National Institute on Aging/Alzheimer’s Association (NIA-AA) [31].

## Methods

### Subjects

We identified patients with a diagnosis of AD who were seen at Taichung Veterans General Hospital between January 2017 and October 2021 retrospectively from the hospital's dementia care database. All enrolled patients were at least 65 years old and clinically diagnosed with AD.

Data collection, including a brief medical history, examination findings, age, sex, body mass index, documented medical comorbidities (including cardiovascular and metabolic diseases), and cigarette and alcohol usage, was conducted by one well-trained licensed nurse case manager. Vascular risk factors, including hypertension, diabetes mellitus, hyperlipidemia, peripheral or cardiac vasculopathy, atrial fibrillation, were gathered to represent comorbid vascular burden of each participant. Each patient's cognitive ability was assessed with a 12-item word recall test (total correct trials 1–3, and 15-min delayed free recall) [32], an estimated Mini-Mental State Examination (MMSE) converted from the Cognitive Abilities Screening Instrument [33, 34], the Montreal Cognitive Assessment (MoCA) [35], and the Clinical Dementia Rating scale (CDR) [36]. Cognitive performance was registered together with diagnostic work-up findings, dementia type, and pharmacological management.

### Clinical diagnosis

Medical records and registry data were reviewed in detail. Dementia diagnoses were based on a clinical interview, functional assessments, neurological examinations, cognitive screening, brain MRI, and blood analyses. Probable AD was diagnosed in accordance with the clinical criteria proposed by the National Institute on Aging/Alzheimer’s Association workgroup [31] as well as the criterion of a global CDR score ≥ 0.5. Non-AD causes of dementia, such as stroke, Parkinson disease, thyroid dysfunction, renal insufficiency, unstable diabetes mellitus, trace element deficiency, and neurosyphilis, were excluded.

### Brain MRI

All subjects were scanned in a 1.5-T MRI scanner (MAGNETOM Aera, Siemens Healthcare, Erlangen, Germany) within 3 months of their cognitive assessment date. The following standardized MR sequences were used: axial spin echo (SE) T1-weighted imaging [repetition time (TR)/echo time (TE) = 550/8.9 ms, field of view (FOV) = 230 × 230 mm^2^, matrix size = 320 × 256, slice thickness = 6 mm), axial turbo-spin echo (TSE) T2-weighted imaging (T2WI) (TR/TE = 3500/103 ms, FOV = 230 × 208 mm^2^, matrix size = 512 × 384, slice thickness = 6 mm), oblique coronal T2WI TSE scan perpendicular to the axis of the hippocampus (TR/TE = 4000/83 ms, FOV = 180 × 180 mm^2^, matrix size = 384 × 307, slice thickness = 3 mm), axial fluid-attenuated inversion recovery (FLAIR)(TR/TE/inversion time = 9000/86/2500 ms, FOV = 230 × 201 mm^2^, matrix size = 320 × 240, slice thickness = 6 mm), axial diffusion weighted imaging (TR/TE = 6300/89 ms, FOV = 230 × 230 mm^2^, matrix size = 192 × 192, slice thickness = 6 mm), axial susceptibility-weighted imaging (SWI) (TR/TE = 49/40 ms, FOV = 230 × 186 mm^2^, flip angle = 15°, matrix size = 288 × 230, slice thickness = 2 mm), and three-dimensional time-of-flight magnetic resonance angiography (TR/TE = 24/7 ms, FOV = 180 × 180 mm^2^, matrix size = 256 × 218, flip angle = 25°, slice thickness = 0.5 mm, slabs = 4 slices per slab = 52). The total examination is about 35 min.

### Visual rating scales

The MRI data were rated by two independent experienced neuroradiologists (Chun-Fu Lin and Hung-Chieh Chen) who were blinded to patient diagnoses. Regional brain atrophy was measured with visual rating scales based on the T1-weighted images as previously described [37]. Medial temporal atrophy (MTA), posterior atrophy (PA), and frontal lobe atrophy were assessed with the MTA scale (range, 0–4) [38], Koedam’s scale (range, 0–3) [39], and global cortical atrophy scale–frontal subscale (GCA-F) (range, 0–3) [40], respectively.

SVD imaging markers were assessed according to the STRIVE consensus [41]. CMBs, recognized as ≤ 10-mm-diameter circular hypointense lesions on SWI, were counted in lobar, deep, and infratentorial regions recorded based on the Microbleed Anatomical Rating Scale (MARS) [42]. CMBs in all brain regions were summed to determine the total CMB burden. Ill-defined hyperintensities ≥ 5 mm across on T2WI and FLAIR images were considered WMH and graded according to the age-related White Matter Change (ARWMC) scale (range, 0–3) in the hemisphere of more severe hyperintensity [43]. ARWMC scores detected in five brain regions (frontal, parietal-occipital, temporal, BG, and infratentorial) were summed to represent total WMH burden. The ARWMC scale has been shown to correlate with WMH volume [44]. A modified Fazekas scale (range, 0–3) was used to grade periventricular and deep WMH [45]. PVSEs were identified when perivascular space regions exceeding 3 mm with signal intensity similar to cerebrospinal fluid on T2WI were visible [46]. PVSEs were counted in the BG and CSO and rated according to quantity as follows: 0, no PVSEs; 1, 1–10; 2, 11–20; 3, 21–40; and 4, > 40 PVSEs. PVSE scores in the BG and CSO were summed to obtain the total PVSE burden. We counted lacunes visible on FLAIR sequence images. Curvilinear hypointensities that followed the gyral surface on SWI were recorded as cortical superficial sideroses (CSSs). Hippocampal sclerosis was defined as an atrophic hippocampus associated with hyperintense signal on long-repetition-time sequences confined to the hippocampus on MRI [47, 48]. The inter-rater reliability for MTA, PA, GCA-F, modified Fazekas scale, MARS total score, ARWMC total score, PVSE total score, and lacune count were between 0.87 and 1.

### AD subtypes based on brain atrophy patterns

Deviation from normality was established following published cutoffs [37]. MTA scores ≥ 1.5, ≥ 1.5, ≥ 2, and ≥ 2.5 were considered abnormal for the respective age ranges of 45–64, 65–74, 75–84, and 85–94 years. A score ≥ 1 was defined as abnormal irrespective of age range because age correction does not improve PA and GCA-F diagnostic performance [37]. Based on the combination of MTA, PA, and GCA-F grades, AD can be classified into four subtypes [1, 8, 26–28, 49] (see Table 1 in Additional file 1). The typical AD subtype was defined as abnormal MTA with abnormal PA and/or abnormal GCA-F. The LP subtype was defined as abnormal MTA with normal PA and GCA-F. The HS subtype was defined as abnormal PA and/or abnormal GCA-F but normal MTA. The MA subtype was defined as normal MTA, PA, and GCA-F.

**Table 1:** Clinical and imaging characteristics of the cohort, *N* = 137

Characteristic	Typical (*N* = 33)		LP (*N* = 26)		HS (*N* = 40)		MA (*N* = 38)		*P* value
Sex, female, *N* (%)	20	(60.6%)	12	(46.2%)	25	(62.5%)	31	(81.6%)	0.030^*^
Age, years	81.0	(74.5–83.5)	76.5	(65.8–84.3)	79.5	(76.0–84.0)	77.0	(70.8–79.0)	0.010^*^
Education, years	6.0	(6–10.5)	6.0	(6.0–12.0)	6.0	(6.0–9.0)	6.0	(0.0–12.0)	0.659
Smoking, *N* (%)	4	(12.1%)	1	(3.8%)	2	(5.0%)	3	(7.9%)	0.588
Vascular risk factors									
Hypertension, *N* (%)	18	(54.5%)	12	(46.2%)	26	(65.0%)	23	(60.5%)	0.466
Diabetes mellitus, *N* (%)	12	(36.4%)	7	(26.9%)	21	(52.5%)	10	(26.3%)	0.067
Hyperlipidemia, *N* (%)	7	(21.2%)	9	(34.6%)	21	(52.5%)	14	(36.8%)	0.053
Peripheral or cardiac vasculopathy^a^	0	(0.0%)	6	(23.1%)	7	(17.5%)	6	(15.8%)	0.052
Atrial fibrillation, *N* (%)	4	(12.1%)	1	(3.8%)	2	(5.0%)	3	(7.9%)	0.588
Number of vascular risk factors	1.0	(0.0–2.0)	0.5	(0.0–3.0)	2.0	(1.0–3.0)	1.0	(0.0–3.0)	0.091
Use of antiplatelet, *N* (%)	5	(15.2%)	5	(19.2%)	13	(32.5%)	13	(34.2%)	0.186
Use of anticoagulant, *N* (%)	3	(9.1%)	1	(3.8%)	2	(5.0%)	2	(5.3%)	0.826
MMSE	18.0	(12.5–21.5)	16.0	(12.5–21.0)	20.0	(16.0–22.8)	20.0	(14.0–22.5)	0.117
MoCA	12.0	(9.5–14.0)	9.0	(6.5–13.5)	14.0	(10.0–18.0)	14.5	(8.3–18.8)	0.045^*^
CDR	1.0	(0.5–1.0)	1.0	(0.8–1.0)	1.0	(0.5–1.0)	0.5	(0.5–1.0)	0.021^*^
CDR—sum of boxes	6.0	(3.6–8.0)	6.0	(4.3–7.5)	4.0	(3.0–5.5)	4.5	(3.0–5.4)	0.006^**^
Hippocampal sclerosis	16	(48.5%)	21	(80.8%)	1	(2.5%)	0	(0.0%)	< 0.001^**^
Cortical superficial siderosis	1	(3.1%)	1	(3.8%)	1	(2.5%)	0	(0.0%)	0.724
CMB	1.0	(0.0–2.0)	0.0	(0.0–1.3)	1.0	(0.0–3.0)	0.0	(0.0–2.0)	0.677
Lobar CMB	0.0	(0.0–1.0)	0.0	(0.0–1.0)	1.0	(0.0–1.5)	0.0	(0.0–1.0)	0.648
Non-lobar CMB	0.0	(0.0–1.0)	0.0	(0.0–1.0)	0.0	(0.0–1.0)	0.0	(0.0–0.5)	0.945
Lacunae	0.0	(0.0–2.0)	0.5	(0.0–3.0)	1.0	(0.0–3.0)	0.0	(0.0–1.0)	0.023^*^
Periventricular WMH	2.0	(1.0–3.0)	2.0	(1.0–2.0)	1.5	(1.0–2.0)	1.0	(1.0–2.0)	0.013^*^
Deep WMH	1.0	(1.0–3.0)	1.5	(1.0–2.0)	1.0	(1.0–2.0)	1.0	(1.0–2.0)	0.248
WMH burden	5.0	(3.0–8.5)	5.0	(2.8–7.3)	5.0	(2.0–7.0)	4.0	(2.0–6.0)	0.298
BG PVSE	3.0	(2.0–3.5)	3.0	(2.0–3.0)	3.0	(2.0–3.0)	2.5	(2.0–3.0)	0.956
CSO PVSE	3.0	(2.5–4.0)	3.0	(2.0–3.0)	3.0	(2.0–4.0)	3.0	(2.0–3.0)	0.381
PVSE burden	6.0	(4.5–7.0)	6.0	(4.0–6.0)	5.0	(4.0–7.0)	5.0	(4.0–6.0)	0.527
Global SVD score	3.0	(2.0–4.0)	2.0	(1.0–3.0)	2.5	(2.0–4.0)	1.0	(1.0–3.0)	0.001^**^
CAA-SVD score	2.0	(1.0–2.0)	1.0	(1.0–2.0)	1.0	(1.0–2.0)	1.0	(0.0–1.0)	0.038^*^
HA-SVD score	2.0	(1.0–3.0)	2.0	(1.0–3.0)	2.0	(1.0–3.0)	1.0	(0.0–2.0)	0.002^**^

Continuous variables, presented as median values and interquartile ranges, were analyzed with the Kruskal–Wallis test; categorical variables, presented as number of patients with percentage, were examined with the chi-square test

*Abbreviations*: *LP* limbic-predominant type, *HS* hippocampal-sparing type, *MA* minimal-atrophy type, *MMSE* mini-mental state examination, *MoCA* Montreal Cognitive Assessment, *CDR* clinical dementia rating, *CMB* cerebral microbleeds, *WMH* white matter hyperintensity, *BG* basal ganglia, *CSO* centrum semiovale, *PVSE* perivascular space enlargement, *SVD* small vessel disease, *CAA* cerebral amyloid angiopathy, *HA* hypertensive arteriopathy

^*^p < 0.05

^**^p < 0.01

^a^Includes carotid artery stenosis, coronary artery disease, myocardial infarction, and peripheral artery disease

### SVD composite scoring

We adopted three validated composite scoring systems based on four established MRI markers of SVD to compile and semi-quantify global SVD severity, CAA-specific pathological burden, and HA-specific pathological burden as a global SVD score, CAA-SVD score, and HA-SVD score, respectively [22, 23, 29, 30]. Briefly, in global SVD scoring (ordinal 0–6 scale), one point (each) was scored for 1–4 lobar CMBs, ≥ 1 lacunes, ≥ 20 BG PVSEs, and moderate WMH (total periventricular + deep WMH grade of 3–4), while 2 points (each) were allocated for ≥ 5 CMBs and severe WMH (total periventricular + deep WMH grade of 5–6). In CAA-SVD scoring (ordinal 0–6 scale), one point (each) was allocated for 2–4 lobar CMBs, ≥ 20 CSO PVSEs, presence of ≥ 2 deep WMH or 3 periventricular WMH or focal CSSs, while 2 points (each) were allocated for ≥ 5 lobar CMBs or disseminated CSSs. In HA-SVD scoring (ordinal 0–4 scale), one point (each) was scored for ≥ 1 deep CMBs, ≥ 1 lacunes, ≥ 10 BG PVSEs, and the presence of ≥ 2 deep WMH or 3 periventricular WMH.

### Z-score generation

*Z*-score was expressed in terms of standard deviation from its mean. The *z* score of each SVD marker measure and cognitive measure of a participant was calculated as follows: *z* = (*x* − *μ*)/*σ* where *x* was the raw score, *μ* was the cohort mean, and *σ* was the cohort standard deviation. Individual *z*-score was computed with the entire group of the enrolled subjects in this study as a reference.

### Composite cognitive score

We generated neurocognitive numeric composite *z*-scores by calculating individual *z*-scores for each test and then averaging them across the cognitive test set. The constituents of the composite *z*-scores were as follows: orientation (orientation tests in the MMSE and MoCA); attention (serial 7 s on the MMSE; sustained attention task; and MoCA forward and backward digit span assessments), short-term memory (STM) registration (total number of items remembered over 3 trials on a 12-item memory test; and MMSE immediate memory task), STM recall (15-min delayed recall on the 12-item memory test; and delayed memory recall tasks in the MMSE and MoCA); language (confrontation naming task, repetition of two syntactically complex sentences, writing, and fluency tasks of the MMSE and MoCA); and visual executive function (MMSE pentagon test; and modified trail making test, clock-drawing task, and three-dimensional cube copy in the MoCA).

### Statistical analysis

Analyses were performed in SPSS version 22.0 for Windows (SPSS Inc., Chicago, IL). Two-tailed *p*-values < 0.05 were considered significant. Differences in continuous/categorical variables between AD subtypes were examined with Kruskal–Wallis test/chi-square tests. Linear regression analysis was completed to detect imaging variable associations with global cognition and constituent cognitive domains after controlling for age, sex, number of vascular risk factors, and CDR. Cognitive and imaging differences among the subtype groups were examined with multivariate general linear models that were controlled for age, sex, vascular risk factors, and CDR, with multiple comparison testing by way of the least significant difference method. To investigate cognitive and imaging variable associations with AD subtypes (reference group: MA subtype), we developed a logistic regression model after adjustment for age, sex, number of vascular risk factors, and CDR. Adjusted odds ratio (OR) values are reported with 95% confidence intervals (CIs). Finally, to examine the synergistic effect of CAA and HA on cognitive performance across subtypes, first, we did receiver operating characteristic and area under the curve analyses to define the CAA-SVD cut-off value for discriminating the MA subtype vs. non-MA subtype (i.e., typical, LP, and HS subtypes), and subsequently, we conducted Pearson correlation coefficient to determine the correlation of HA-SVD score with domain-specific cognitive performance between CAA-SVD score ≤ 1 vs. > 1 across AD subtypes (see Additional file 1).

## Results

### Clinical and imaging characteristics of the entire cohort

**Fig. 1:**
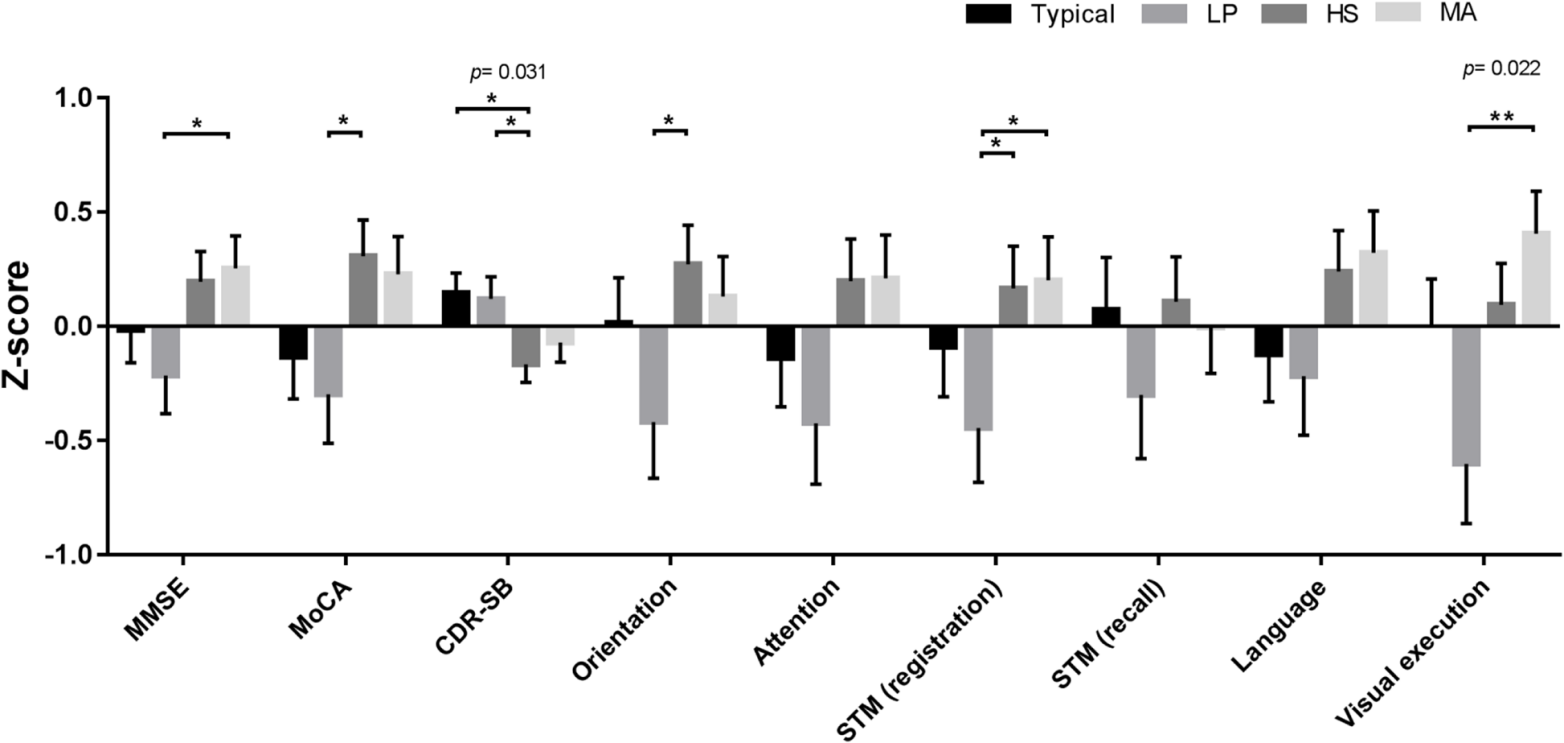
Relation of cognitive measures to AD subtypes The bar graph depicts z-scores (mean and standard error) of cognitive measures. *P*-values indicate the main effect of subtype grouping on the respective scores in multivariate general linear models controlled for age, sex, vascular risk factors, and CDR with multiple comparison tested by least significant difference; *p < 0.05. *LP *limbic-predominant type, *HS*, hippocampal-sparing type; *MA*, minimal-atrophy type, *MMSE*, mini-mental state examination, *MoCA* Montreal cognitive assessment, *CDR-SB* clinical dementia rating-sum of boxes, *STM* short-term memory

The main demographic and imaging characteristics of the study cohort are summarized in Table 1. A total of 137 patients with AD were classified into four groups: typical subtype (*N* = 33), LP subtype (*N* = 26), HS subtype (*N* = 40), and MA subtype (*N* = 38). The median duration between MRI testing and cognitive evaluation was 1.1 months. The four subtype groups differed significantly with respect to age, sex, MoCA score, CDR scale, and sum of boxes of CDR (CDR-SB) score. Additionally, the groups differed significantly with respect to the imaging prevalence of hippocampal sclerosis, lacune amount, periventricular WMH, composite global SVD scores, composite CAA-SVD scores, and composite HA-SVD scores. All enrolled subjects did not fulfill the modified Boston criteria for CAA [50]. Correlations between composite cognitive scores were examined (see Fig. 1 in Additional file 1).

**Fig. 2:**
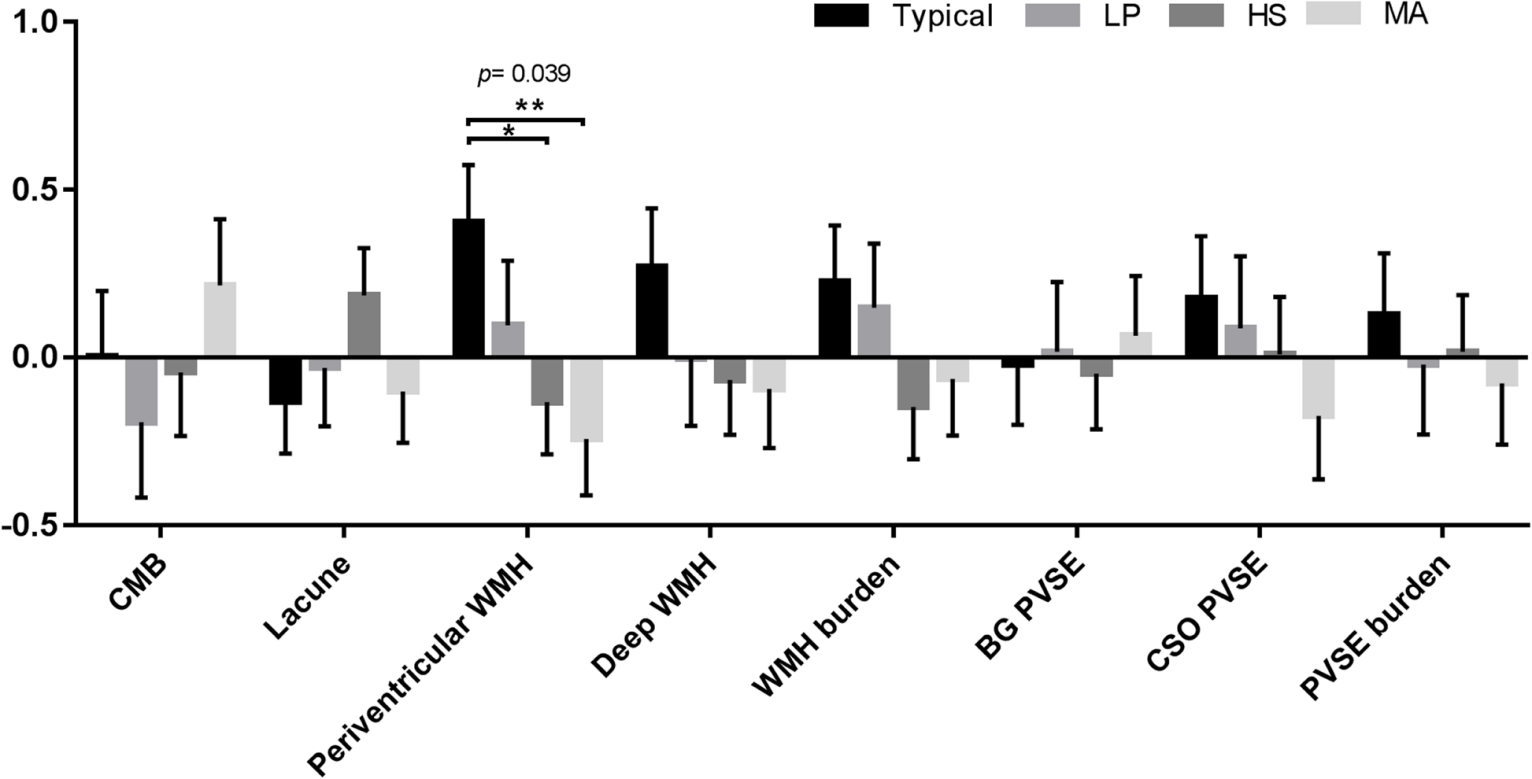
Relation of singular imaging markers to AD subtypes The bar graph depicts z-scores (mean and standard error) of marker measures. *P*-values indicate the main effect of subtype grouping on the respective imaging visual rating scores in multivariate general linear models controlled for age, sex, vascular risk factors, and CDR with multiple comparison tested by least significant difference; *p <0.05. *LP* limbic-predominant type, *HS* hippocampal-sparing type, *MA* minimal-atrophy type, *CMB* cerebral microbleeds, *WMH* white matter hyperintensity, *BG* basal ganglia, *CSO* centrum semiovale, *PVSE* perivascular space enlargement

**Fig. 3:**
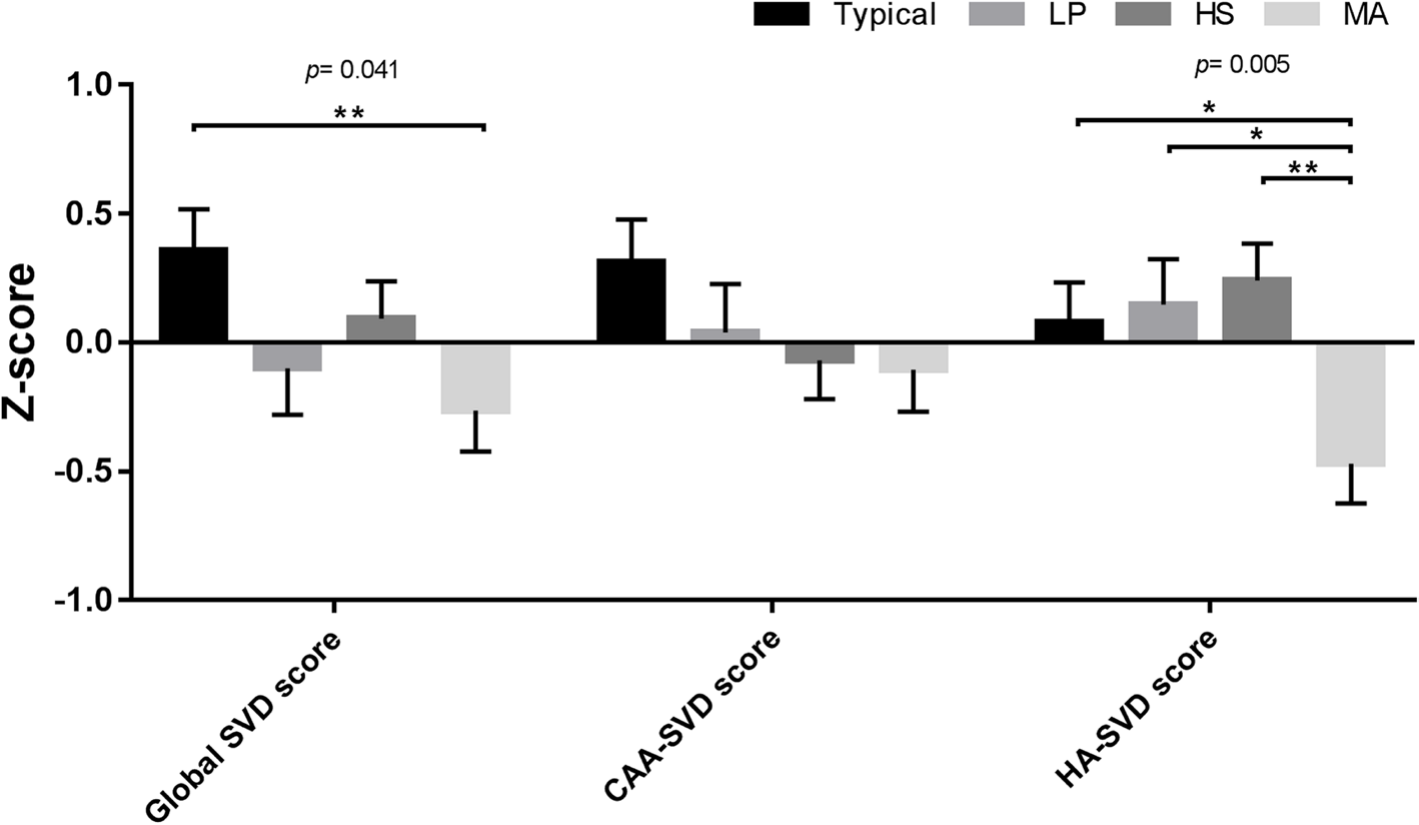
Relation of composite imaging markers to AD subtypes The bar graph depicts z-scores (mean and standard error) of composite imaging measures. *P*-values indicate the main effect of subtype grouping on each composite imaging visual rating score using multivariate general linear models controlled for age, sex, vascular risk factors, and CDR with multiple comparison tested by least significant difference; *p < 0.05. *LP* limbic-predominant type, *HS* hippocampal-sparing type, *MA* minimal-atrophy type, *SVD* small vessel disease, *CAA* cerebral amyloid angiopathy, *HA* hypertensive arteriopathy

Figures 1, 2, and 3 displayed the cognitive and imaging differences across the subtype groups using multivariate general linear models controlled for age, sex, vascular risk factors, and CDR, with multiple comparison testing by way of the least significant difference method. In Fig. 1, there were significant differences in CDR-SB and visual executive function among 4 subtypes. MoCA, CDR-SB, orientation, and STM (registration) differed significantly between the LP and HS subtypes, while MMSE, STM (registration), and visual executive function differed significantly between the LP and MA subtypes. Also, CDR-SB significantly differed between the typical and HS subtypes.

SVD marker distributions across the AD subtypes are presented in Fig. 2 and Fig. 3. The typical subtype was the subtype with the greatest periventricular WMH presence and global SVD scores, whereas the MA subtype group had the lowest periventricular WMH presence, global SVD scores, and HA-SVD scores. Meanwhile, we found that the subtype groups had differing distribution patterns of the three composite scores along the spectrum of CAA and HA (Fig. 3). The global SVD scores obtained for the LP and HS subtypes were intermediate between the typical and MA subtypes. CAA-SVD scores showed a decreasing trend in the order of typical, LP, HS, and MA subtypes. Of the four groups, the MA subtype group had the lowest HA-SVD scores. The three scores reflective of two distinct underlying pathophysiological processes in CAA and HA yielded differential associations with the four AD subtypes.

### Associations of imaging variables with global cognition and cognitive domains

Linear regression analysis adjusted for age, sex, vascular risk factors, and CDR (Table 2) showed that deep WMH presence correlated with poorer STM (registration and recall). All three composite SVD scores correlated with lower global cognition (i.e., lower MMSE score). Additionally, global SVD scores correlated with higher global disease severity (i.e., higher CDR-SB score). CAA-SVD scores correlated with poorer performance on STM (registration) while HA-SVD scores correlated with lower global cognition (i.e., lower MoCA score) and poorer orientation, attention, language, and visual executive functions.

**Table 2:** Correlations of singular imaging variables with neurocognitive domains, *N* = 137

Cognitive domain	CMB	Lacune	PV WMH	Deep WMH	WMH burden	BG PVSE	CSO PVSE	PVSE burden	Global SVD score	CAA-SVD score	HA-SVD score
	***β***	***β***	***β***	***β***	***β***	***β***	***β***	***β***	***β***	***β***	***β***
MMSE	− .009	− .027	− .006	.015	.025	.017	.026	.064	− .064^*^	− .044^**^	− .115^**^
MoCA	.094	.047	− .003	.020	.022	.017	.002	.034	.001	− .012	− .068^**^
CDR-SB	.401	.122	.062	.023	.038	.013	.036	.012	.212^*^	.043	.045
Orientation	− .047	− .011	− .038	.009	− .074	.101	− .073	− .070	− .173	− .054	− .297^**^
Attention	.571	.027	− .060	.029	− .089	.024	− .002	.080	− .024	− .165	− .411^**^
STM (registration)	− .431	.251	− .092	− .207^*^	− .398	.001	− .105	.023	− .255	− .219^**^	− .208
STM (recall)	− .304	.024	− .036	− .193^*^	− .417	.005	.105	.168	− .232	− .009	− .021
Language	.188	.304	− .080	.048	.027	− .097	− .115	− .125	− .214	− .200^*^	− .429^**^
Visual execution	.463	.176	.006	.063	.321	.101	− .016	.117	.127	− .042	− .403^**^

Linear regression, adjusted for age, sex, vascular risk factors, and CDR, was calculated to explore correlations between cognitive and imaging variable

*Abbreviations*: *CMB* cerebral microbleeds, *PV* periventricular, *WMH* white matter hyperintensity, *BG* basal ganglia, *CSO* centrum semiovale, *PVSE* perivascular space enlargement, *SVD* small vessel disease, *CAA* cerebral amyloid angiopathy, *HA* hypertensive arteriopathy, *MMSE* mini-mental state examination, *MoCA* Montreal Cognitive Assessment, *CDR* clinical dementia rating score, *CDR-SB* clinical dementia rating-sum of boxes, *STM* short-term memory

^*^p < 0.05,

^**^p < 0.01

### Associations of cognitive and imaging variables with AD subtypes

The relationship between cognitive and imaging variables and AD subtypes was examined through a logistic regression analysis controlled for age, sex, and vascular risk factors, and CDR (Table 3). Typical subtype was found to be positively associated with periventricular WMH (OR 2.62; 95%CI, 1.23–5.57, *p* = 0.012), global SVD score (OR 1.67; 95%CI, 1.11–2.52, *p* = 0.009), and HA-SVD score (OR 1.93; 95%CI, 1.10–3.52, *p* = 0.034). LP subtype was found to be positively associated with HA-SVD score (OR 2.57; 95%CI, 1.23–5.37, *p* = 0.012) but negatively associated with all global and domain-specific cognitive scores, except for CDR-SB and STM (recall). HS subtype was found to be positively associated with HA-SVD score (OR 3.30; 95%CI, 1.58–6.85, *p* = 0.001).

**Table 3:** Associations of neurocognitive domains and imaging variables with AD subtypes (MA subtype as the reference).

Domain/Variable	Typical			LP			HS		
	OR	95%CI	*P *value	OR	95%CI	*P *value	OR	95%CI	*P *value
MMSE	0.96	(0.85–1.09)	0.514	0.82	(0.69–0.96)	0.015*	1.02	(0.90–1.15)	0.750
MoCA	0.92	(0.80–1.06)	0.259	0.76	(0.61–0.94)	0.014*	1.04	(0.92–1.19)	0.486
CDR-SB	1.27	(0.84–1.91)	0.254	1.59	(0.93–2.71)	0.086	0.77	(0.46–1.27)	0.317
Orientation	0.75	(0.33–1.69)	0.492	0.18	(0.05–0.70)	0.013*	1.17	(0.60–2.30)	0.640
Attention	0.69	(0.34–1.41)	0.307	0.15	(0.04–0.52)	0.003**	1.07	(0.55–2.07)	0.838
STM (registration)	0.61	(0.27–1.36)	0.225	0.26	(0.10–0.70)	0.007**	1.07	(0.57–2.01)	0.823
STM (recall)	0.977	(0.51–1.86)	0.944	0.47	(0.17–1.28)	0.141	1.12	(0.61–2.04)	0.704
Language	0.62	(0.29–1.30)	0.207	0.21	(0.07–0.66)	0.008**	0.97	(0.48–1.99)	0.948
Visual execution	0.68	(0.33–1.39)	0.288	0.12	(0.03–0.54)	0.006**	0.77	(0.40–1.51)	0.456
CMB	0.98	(0.87–1.10)	0.806	0.88	(0.69–1.12)	0.297	0.96	(0.83–1.11)	0.590
Lacune	1.01	(0.75–1.34	0.961	1.04	(0.75–1.44)	0.819	1.24	(0.93–1.68)	0.147
Periventricular WMH	2.62	(1.23–5.57)	0.012*	2.06	(0.85–4.97)	0.108	1.22	(0.63–2.33)	0.549
Deep WMH	1.65	(0.93–2.96	0.089	1.18	(0.61–2.30)	0.630	1.17	(0.68–2.00)	0.555
WMH burden	1.11	(0.94–1.32)	0.244	1.14	(0.93–1.40)	0.200	0.994	(0.82–1.20)	0.947
BG PVSE	1.03	(0.57–1.87)	0.914	0.99	(0.49–1.99)	0.988	0.74	(0.39–1.37)	0.340
CSO PVSE	1.45	(0.84–2.50)	0.182	1.24	(0.65–2.40)	0.516	1.13	(0.67–1.89)	0.635
PVSE burden	1.25	(0.86–1.80)	0.237	1.02	(0.70–1.47)	0.924	1.03	(0.72–1.47)	0.850
Global SVD score	1.67	(1.11–2.52)	0.009**	1.16	(0.75–1.79)	0.502	1.23	(0.85–1.78)	0.269
CAA-SVD score	1.53	(0.79–2.95)	0.204	1.14	(0.56–2.33)	0.767	1.09	(0.63–1.90)	0.761
HA-SVD score	1.93	(1.10–3.52)	0.034*	2.57	(1.23–5.37)	0.012*	3.30	(1.58–6.85)	0.001**

ORs determined by multivariate logistic regression, adjusted for age, sex, vascular risk factors, and CDR

*LP* limbic-predominant type, *HS* hippocampal-sparing type, *MA* minimal-atrophy type, *MMSE* mini-mental state examination, *MoCA* Montreal Cognitive Assessment, *CDR* clinical dementia rating score, *CDR-SB* clinical dementia rating-sum of boxes, *STM* short-term memory, *CMB* cerebral microbleeds, *WMH* white matter hyperintensity, *BG* basal ganglia, *CSO* centrum semiovale, *PVSE* perivascular space enlargement, *SVD* small vessel disease, *CAA* cerebral amyloid angiopathy, *HA* hypertensive arteriopathy

^*^p < 0.05

^**^p < 0.01

### Synergistic effects of CAA and HA across AD subtypes

Pearson correlation coefficient revealed that HA-SVD score correlates differently with cognitive performance between CAA-SVD > 1 vs. CAA-SVD score ≤ 1 in each atrophic subtype (see Table 2 and Table 3 in Additional file 1).

## Discussion

This retrospective cohort study of clinically diagnosed late-onset AD examined the role of SVD across AD subtypes and their clinical implications. We found that the four examined AD subtypes had comparable clinical vascular risk factor burdens. The typical and LP subtype groups had more cognitive impairment and greater dementia severity than the MA subtype group. Regarding singular SVD imaging markers, only periventricular WMH was highest in the typical subtype group and deep WMH correlated with poor STM. The three composite scores correlated differentially with cognitive domain performance and were found to have differential associations with the four AD subtypes. In reference to the MA subtype, the typical subtype group had the most periventricular WMH, the greatest disease severity, the highest global SVD burden, and high HA-SVD scores. The LP subtype group had extensive cognitive impairment and high HA-SVD scores. The HS subtype group had high HA-SVD scores. Compared to singular MRI markers, the composite scores, which include CAA and HA SVD contributions, correlated more robustly with global and domain-specific cognitive function performance and associated more distinctly with AD subtypes. Each subtype group exhibited a distinctive pattern of the three composite scores over the spectrum of CAA and HA. Of the three, HA-SVD score was the most essential in determining cognitive consequences. CAA and HA seem to pose differential synergistic effects on cognitive performance and atrophic patterns in AD. To the best of our knowledge, the current study is the first to report domain-specific cognitive associations of comprehensive SVD imaging markers as well as data pointing to their implications across subtypes.

The prevalence of AD subtypes varied across studies [1, 5, 6]. A meta-analysis of 24 studies examined the pooled frequency of the four subtypes, showing that typical subtype is most common, with a pooled frequency of 55% [1]. LP, HS, and MA subtypes had a pooled frequency of 21, 17, and 15%, respectively [1]. The prevalence rates of subtypes in our cohort were discordant with the previous finding might be partially explained by different cohort characteristics and sample size.

Although it may be argued that the four subtypes reflect AD patients at different stages of the disease, rather than exactly distinct AD subtypes, it has been demonstrated that these subtypes indeed have differential spread of neurofibrillary tangles in the circumstance of similar disease duration and disease severity staged by CDR score [3]. Likewise, evidence has shown that there are no differences in CDR or disease duration in the MA subtype, suggesting that this subtype might not be an initial stage of AD [6, 8]. Alternatively, it may be argued that visual rating scales might not be sensitive enough to detect minimal atrophy in the MA subtype, but lack of atrophy has been confirmed in the MA subtype in previous studies using automated MRI method and voxel-based morphometry [8, 51].

Numerous differences have been identified across AD subtypes [1, 5, 6]. Several clinical features of the 4 subtypes have been well described. The typical subtype is prone to be old age of onset, multidomain amnestic syndrome, low final MMSE score, high burden of WMH and CAA, and frequent APOE ε4 carrier [1, 5, 6]. The LP subtype is apt to be old age at onset/death, female sex, amnestic syndrome, slow disease progression, higher burden of WMH, frequent APOE ε4 and MAPT H1H1 genotypes, hippocampal sclerosis, and more HA but less CAA [1, 5, 6]. The HS subtype tends to be young age of onset/death, male sex, nonamnestic syndrome, faster disease progression, APOE ε4 noncarrier, low WMH burden, and more CAA, but less HA [1, 5, 6]. The MA subtype has intermediate/young age of onset, slow cognitive decline, and tendency to be misdiagnosed as normal old or other dementia syndromes [1, 5, 6]. In line with the above-mentioned characteristics of each subtype, our typical subtype patients tended to be older; typical and LP subtype patients often had amnestic presentations; LP subtype patients often had hippocampal sclerosis; typical subtype patients had a prominent WMH burden and a strong HA influence.

CAA and HA pathologies, which are common in late-onset AD [13, 52], may modify AD progression or aggravate risk by shifting the threshold for cognitive impairment and dementia. The contribution of SVD to AD pathogenesis has been reviewed in detail elsewhere [15, 53, 54]. The mechanisms by which CAA and HA promote AD pathology may involve chronic cerebral hypoperfusion or BBB dysfunction. The former could cause white matter neurodegeneration and worsen AD pathology (β-amyloid and tau), while the latter could impede vascular clearance path through the BBB and glymphatic system; furthermore, aggregated neurotoxic peptides lead to ischemic neuronal death [15, 53, 54]. Our findings might support that clinical AD subtyping may be partially the consequence of differing CAA and HA burdens in the AD brains. Although the exact interactions between vascular and neurodegenerative processes in AD are not completely understood, SVD changes precede β-amyloid deposition over long periods of time and can predict disease progression [53, 55].

Of all the markers assessed here, deep WMH burden and the three composite scores were associated with cognition, suggesting that different MRI markers of vascular brain injury have differing cognitive significance. Of singular imaging markers, we found that only periventricular WMH is more pervasive in the typical subtype than in the MA subtype. Studies have shown that WMH could predate dementia symptoms in both autosomal dominant AD and late-onset AD [56–58]. In late-onset AD, WMH are commonly present in periventricular and deep regions and a high WMH burden has been related to poor clinical and cognitive outcomes [15, 59]. WMH pathology correlates with demyelination, axonal abnormalities, pericyte cell loss, hemosiderin deposition, arteriolosclerosis, and BBB dysfunction of vascular origin as well as with degenerative axonal loss (Wallerian degeneration) secondary to the deposition of cortical AD pathology of non-vascular origin [60, 61]. The presence of concomitant cortical AD pathology and varying CAA and/or HA severity leads to a dissociation between AD diagnosis and SVD imaging makers [15]. Of singular imaging markers, a previous study showed that typical subtype is associated with a relatively high burden of lobar CMB, WMH, and CSO PVSE among clinically diagnosed AD patients [27]. MA subtype had the highest prevalence of probable CAA across subtypes [27]. Their findings suggested that CAA pathology may contribute particularly to the MA subtype, while HA pathology contributes particularly to the typical and LP subtypes. The differences in the findings between our study and their study may be attributed to varying SVD imaging variables, methodological approaches, cohort age, and traditional vascular risk factor burden.

Regarding composite markers, we found that global SVD MRI burden contributed to the typical subtype while HA-specific MRI burden contributed to the typical, LP, and HS subtypes. We found no significant difference in CAA-specific MRI burden across subtypes, although previous pathological and imaging investigations have revealed that pathological burdens and imaging markers of CAA were topographically increased in the lobar regions, particularly with occipital/posterior predominance of CMB and cerebral amyloidosis [20, 62, 63]. We did observe a descending trend from typical, to LP, to HS, to MA subtype, which might suggest that vascular amyloid pathology should have been present in the dementia stage of AD represented by our cohort, irrespective of subtype. Our findings in Fig. 3 and Table 3 suggest that the typical subtype has predominantly high HA burdens, relatively high CAA burdens, and heavy global SVD burdens as compared to other subtypes, which was different from the result of a previous study [27], showing typical and MA subtypes being at opposite ends in a hypothetical continuum of HA and CAA. The disparate finding could be attributable in part to differences in comorbid vascular burden, age, and cohort size. On the basis of our and the previous imaging findings, we infer that (1) CAA burden is prevalent in the dementia stage of AD regardless of subtype and (2) both HA and CAA potentially play synergistic roles in AD subtyping with respect to cognition and atrophic patterns. Our findings reinforce the concept that different cerebrovascular burdens and neurodegeneration patterns may reflect distinct AD subtypes [8, 27, 64].

Individual SVD markers (i.e., microbleed, WMH, lacune, and PVSE) have been related to cognitive function [24, 65–67]. Our composite SVD scores encompassing both CAA and HA not only varied differentially with AD subtypes but also correlated with domain-specific cognitive functions more strongly than singular markers. Global SVD scoring and HA-SVD scoring appear to be more associated with non-MA subtypes than the MA subtype, while HA-SVD scoring seems to be the most essential for determining cognitive consequences. Further research is needed to clarify the exact interactions of amyloid and nonamyloid vasculopathies in the SVD spectrum and their contributions to neurodegenerative processes in AD [19]. Indeed, total burden of coexisting vascular pathological abnormalities, rather than any single lesion type, is the most relevant determinant of cognitive impairment and might offer a window into differing vascular and neurodegenerative interactions across AD subtypes.

The four subtypes represented in this study reflect consistently categorized patterns of brain atrophy in MRI studies [1, 8, 26–28, 49], confirming their usefulness for examining AD subtyping in routine practice. The differential constellations of underlying SVD pathologies could, at least in part, underlie the different patterns of neurodegeneration observed across these subtypes. Different subtypes have varying age of onset/death, clinical manifestations, prevalence of APOE ε4 carriers, depositions of amyloid and tau, vascular burdens of HA and/or CAA, and progression rate [1, 5, 6]. These results imply that (1) early identification and diagnosis of AD subtypes are clinically relevant and the basis for further proper managements since distinct subtypes have different cognitive features and clinical prognoses, (2) differing preventive interventions, disease-modifying therapies, and treatment strategies for different subtypes are urgently needed to be explored and implemented, and (3) therapeutic strategies for SVD should be more specifically tailored since SVD burdens of HA and/or CAA vary across subtypes. Finally, we suggest that applying this MRI-based AD subtyping in clinical trials and practice as well as integrating SVD markers into the ATX(N) classification scheme for AD biomarkers may help to improve the framing of hypotheses, study designs, and the elucidation of mechanisms in subtype and SVD studies [68, 69].

### Limitations of the study

The cohort consisted of consecutive patients diagnosed with AD based only on clinical information. The lack of a biomarker-based scheme for AD diagnosis remains a clinical challenge. A diagnostic accuracy was reported to range from 70 to 80% for a clinical diagnosis of AD [70]. Possibly, some AD-like dementia syndromes might be included in our cohort, such as primary age-related tauopathy, tangle-predominant dementia, limbic-predominant age-related TDP-43 encephalopathy, argyrophilic grain disease, frontotemporal dementia, or Lewy body disease. Despite this potential limitation, the primary motif of this study was to provide information relevant to the interactions between SVD and atrophic patterns in clinically diagnosed AD. Because of lack of lobe-specific distributions of individual imaging variables in this AD subtyping classification system based on the atrophic severities of medial temporal, parietal, and frontal lobes, we might not be able to detect differences in CAA-specific MRI burden across subtypes. Additionally, we used a retrospective and cross-sectional cohort. Longitudinal studies are needed to clarify progression over time. We did not collect comprehensive neuropsychological assessments. Finally, although identifying AD subtypes and semi-quantifying SVD burden on MRI based on visual rating scales should be clinically informative, it remains to be determined whether implementing these methods in clinical settings would improve diagnosis and prognosis for AD patients.

## Conclusions of the study

Our findings add extensive information on singular and composite MRI-based SVD markers and provide insight into CAA and HA synergistic effects on cognitive domains and brain atrophy patterns in AD. Despite CAA burden being homogeneous across the subtypes, HA and CAA differentiate AD subtyping synergistically, with contribution of both CAA and HA to the typical subtype, while HA contributes more to the LP and HS subtypes than to the MA subtype. Our data suggest that the different subtypes could potentially have distinct vascular contributions. If so, distinct combinations of clinical features and concomitant cerebrovascular pathologies may inform treatment planning. Incorporating both MRI-based subtyping and SVD scoring in clinical research and AD patient care may, in addition to elucidating the complex vascular pathogenic mechanisms in the AD spectrum, contribute to the future individualization of patient-tailored therapeutic strategies for addressing vascular contributions.

## Supplementary Information

**Additional file 1:Supplementary Table 1.** Subtypes of AD based on patterns of brain atrophy from visual rating scales. **Supplementary Table 2.**  ROC analysis for CAA-SVD score in differentiating the MA subtype from non-MA subtypes. **Supplementary Table 3.** Associations of HA-SVD scores with composite cognitive scores between patients with CAA-SVD score ≤ 1 vs. >1 across AD subtypes. **Supplementary Figure 1.** The heatmap of the correlation matrix across composite cognitive scores using Pearson correlation coefficient.

## Data Availability

The datasets used and analyzed during the current study are available from the corresponding author on reasonable request.

## Abbreviations

AD: Alzheimer disease
ARWMC: Age-Related White Matter Change scale
BG: Basal ganglia
CAA: Cerebral amyloid angiopathy
CDR: Clinical dementia rating
CDR-SB: Clinical dementia rating-sum of boxes
CMB: Cerebral microbleeds
CSO: Centrum semiovale
CSS: Cortical superficial siderosis
GCA-F: Global cortical atrophy scale—frontal subscale
HA: Hypertensive arteriopathy
HS: Hippocampal-sparing type
LP: Limbic-predominant type
MA: Minimal-atrophy type
MARS: Microbleed Anatomical Rating Scale
MMSE: Mini-mental state examination
MoCA: Montreal Cognitive Assessment
MRI: Magnetic resonance imaging
MTA: Medial temporal atrophy
PA: Posterior atrophy
PVSE: Perivascular space enlargement
STM: Short-term memory
SVD: Small vessel disease
WMH: White matter hyperintensity
