# Supplementary material for: Imaging markers of cerebral amyloid angiopathy and hypertensive arteriopathy differentiate Alzheimer disease subtypes synergistically
Source: Alzheimers Res Ther. 2022 Sep 30;14:141. doi: 10.1186/s13195-022-01083-8 (PMC9524061; doi:10.1186/s13195-022-01083-8)
Supplement: Supplementary file 1 — Additional file 1:Supplementary Table 1. Subtypes of AD based on patterns of brain atrophy from visual rating scales. Supplementary Table 2. ROC analysis for CAA-SVD score in differentiating the MA subtype from non-MA subtypes. Supplementary Table 3. Associations of HA-SVD scores with composite cognitive scores between patients with CAA-SVD score ≤ 1 vs. >1 across AD subtypes. Supplementary Figure 1. The heatmap of the correlation matrix across composite cognitive scores using Pearson correlation coefficient. [file 13195_2022_1083_MOESM1_ESM.docx]

**Additional file 1**

**Supplementary Table 1.** Subtypes of AD based on patterns of brain atrophy from visual rating scales.

|  | | MTA normal | MTA abnormal |
| --- | --- | --- | --- |
| PA normal | GCA-F normal | **MA subtype** | **LP subtype** |
| PA normal | GCA-F abnormal | **HS subtype** | **Typical subtype** |
| PA abnormal | GCA-F normal |  |  |
| PA abnormal | GCA-F abnormal |  |  |

**Synergistic effects of CAA and HA on AD subtypes**

First, we did ROC analysis to determine CAA-SVD cut-off value, yielding the following optimal significant cut-off values for the differentiation of MA subtype and non-MA subtype (i.e., typical, LP, HS subtypes) in Supplementary Table 2.

| **Supplementary Table 2**. ROC analysis for CAA-SVD score in differentiating the MA subtype from non-MA subtypes | | | | | | | |
| --- | --- | --- | --- | --- | --- | --- | --- |
| Variable | AUC | (95% CI) | | *p* | Optimal cutoff | Sensitivity (%) | Specificity (%) |
| CAA-SVD | 0.63 | (0.55- | 0.72) | 0.009 | >1 | 46.46% | 78.95% |
|  |  |  |  |  |  |  |  |

Second, in Supplementary Table 3, Pearson correlation coefficient was used to determine the correlation of HA-SVD score with domain-specific cognitive performance between patients with CAA-SVD score ≤ 1 vs. >1 across AD subtypes. This analysis showed that HA-SVD score correlates differently with cognitive performance between CAA-SVD >1 vs. CAA-SVD score ≤ 1 in each atrophic subtype, suggesting that to some extent differing synergistic effects of HA and CAA occur in different atrophic AD subtypes.

| **Supplementary Table 3**. Associations of HA-SVD scores with composite cognitive scores  between patients with CAA-SVD score ≤ 1 vs. >1 across AD subtypes | | | | | | | | |
| --- | --- | --- | --- | --- | --- | --- | --- | --- |
| Cognitive domains | Typical | | LP | | HS | | MA | |
|  | CAA≤1 | CAA>1 | CAA≤1 | CAA>1 | CAA≤1 | CAA>1 | CAA≤1 | CAA>1 |
| MMSE | .288 | -.621** | .240 | -.167 | -.567** | -.784 | -.857** | -.763* |
| MoCA | -.117 | .388 | -.757* | -.612 | -.445 | -.568* | -.759** | -.888* |
| CDR-SB | -.110 | .530* | -.128 | .300 | .248 | .186 | .253 | .444 |
| Orientation | .241 | -.102 | -.225 | -.899 | -.376 | -.630 | -.642** | .464 |
| Attention | -.025 | -.103 | -.048 | -.908 | -.370 | -.686* | -.697** | -.881* |
| STM (registration) | -.281 | .370 | -.242 | -.569 | -.294 | -.396 | -.569** | -.736 |
| STM (recall) | -.096 | -.772 | -.074 | -.513 | -.159 | -.670* | -.388* | .422 |
| Language | .114 | -.102 | -.011 | -.942 | -.503* | -.674* | -.639** | -.884* |
| Visual execution | -.397 | .226 | -.024 | -.866 | -.566* | -.391 | -.632** | -.919* |
| *p<0.05, **p<0.01. | | | | | | | | |

**Supplementary Figure 1.** The heatmap of the correlation matrix across composite cognitive scores using Pearson correlation coefficient

**
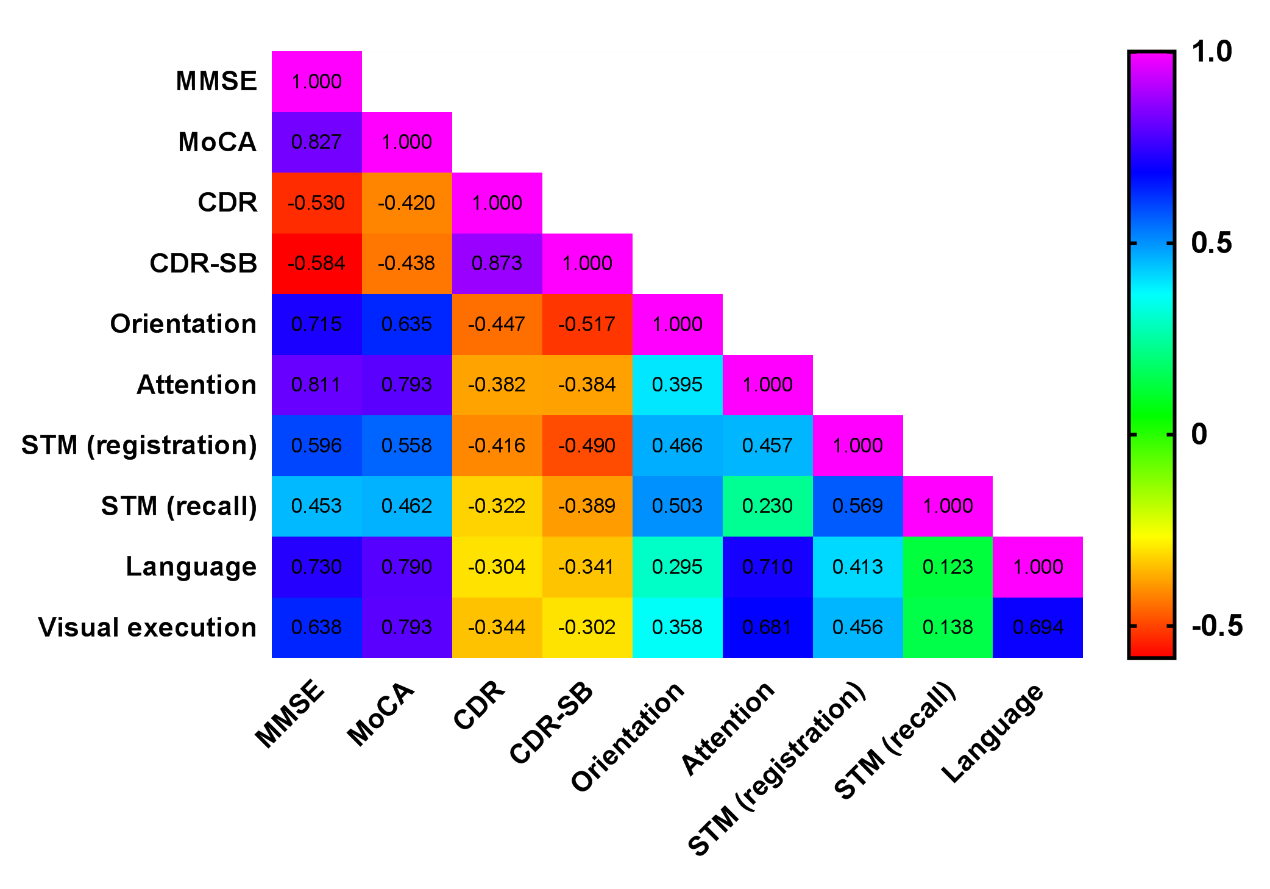
**
